# Crystal structure and Hirshfeld surface analysis of lithium chloride and lithium bromide with dimethyl ether ligands

**DOI:** 10.1107/S2056989025009119

**Published:** 2025-10-31

**Authors:** Julius Hättasch, Annika Schmidt, Carsten Strohmann

**Affiliations:** a Technische Universität Dortmund, Fakultät fü Chemie und Chemische Biologie, Otto-Hahn-Strasse 6, 44227 Dortmund, Germany; National Taras Shevchenko University of Kyiv, Ukraine

**Keywords:** crystal structure, lithium chloride, lithium bromide, dimethyl ether, Hirshfeld surface analysis, lithium halide dimers, tetrel bonds

## Abstract

Lithium chloride and bromide dimethyl ether adducts, di-μ-chlorido-bis­[bis­(dimethyl ether-κ*O*)lithium], [Li_2_Cl_2_(DME)_4_] (**1**), and di-μ-bromido-bis­[bis­(dimethyl ether-κ*O*)lithium], [Li_2_Br_2_(DME)_4_] (**2**) [DME is dimethyl ether, C_2_H_6_O], have been characterized by single-crystal X-ray diffraction. Both com­pounds crystallize as dimers, in which the lithium ions are tetra­hedrally coordinated by two μ-halide ions and two O-centres from the DME ligands. In **1**, the dimers form two-dimensional layers defined by CH_3_⋯Cl tetrel bonds, while the bromide analogue assembles into planar sheets featuring CH_3_⋯CH_3_ contacts. Hirshfeld surface analyses reveal that H⋯H and halogen–hy­dro­gen inter­actions dominate the inter­molecular contacts. The results demonstrate that even the simplest ether, dimethyl ether, can act as an effective coordinating ligand toward lithium halides and influence their aggregation and supra­molecular organization. Thereby, this study explores new advances into the preparation and handling of sophisticated coordination com­pounds with gaseous ligands.

## Abstract 2

The lithium halide dimethyl ether (DME) adducts [Li_2_Cl_2_(DME)_4_] and [Li_2_Br_2_(DME)_4_] form dimeric units linked into layers by CH_3_⋯Cl tetrel bonds or CH_3_⋯CH_3_, CH_3_⋯O and CH_3_⋯Br contacts, illustrating the coordination ability of dimethyl ether and its role in directing supra­molecular assembly.

## 1. Chemical context

Lithium halides have versatile applications in organic synthesis and catalysis. Lithium chloride (LiCl) and lithium bromide (LiBr), for example, can be used as additives to accelerate reaction rates and manipulate regio- and stereoselectivity of Diels–Alder reactions (Arseniyadis *et al.*, 1994 ▸; Oh & Rally, 1994 ▸; Reddy *et al.*, 2021 ▸). Additionally, LiCl has been reported to accelerate li­thia­tion reactions (Gupta *et al.*, 2009 ▸; Henderson *et al.*, 1996 ▸; Knauer & Strohmann, 2020 ▸) and to improve the efficiency of Grignard reagents by modulating solubility and reaction kinetics (Hermann *et al.*, 2023 ▸; Krasovskiy & Knochel, 2004 ▸). Furthermore, both LiCl and LiBr are known to enhance the reducing power of samarium(II) iodide (SmI_2_), making them valuable tools for reductive processes (Fuchs *et al.*, 1997 ▸).

Dimethyl ether is the simplest ether with only two C atoms and has a low boiling point (248.8 ± 1.0 K), which is why it is not often used as a classic solvent. Instead, some of its uses include serving as an alternative to conventional fuels and as an extraction solvent (Zheng & Watanabe, 2022 ▸; Catizzone *et al.*, 2021 ▸). However, there is an absence of structures with this simplest ether, likely due to its difficult handling.

The aggregation state of lithium halides can vary depending on the ligands used in the solid state. For example, single-crystal X-ray studies have shown that LiCl exists as tetra­meric [Li_4_Cl_4_] units in diethyl ether with each lithium ion being bonded with a single ether mol­ecule (Mitzel & Lustig, 2001 ▸). In contrast, our crystallographic investigations reveal that both lithium chloride and lithium bromide form dimeric [Li_2_*X*_2_] units in dimethyl ether (DME), where each lithium ion is bonded with two DME mol­ecules. These findings provide new insights into the influence of ligands on the aggregation behavior of lithium halides.
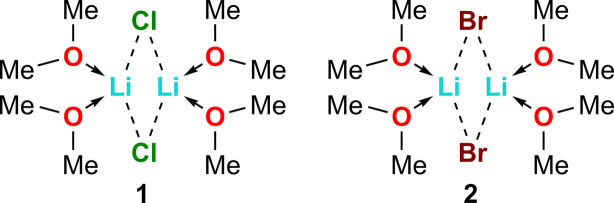


## 2. Structural commentary

**Figure 1:**
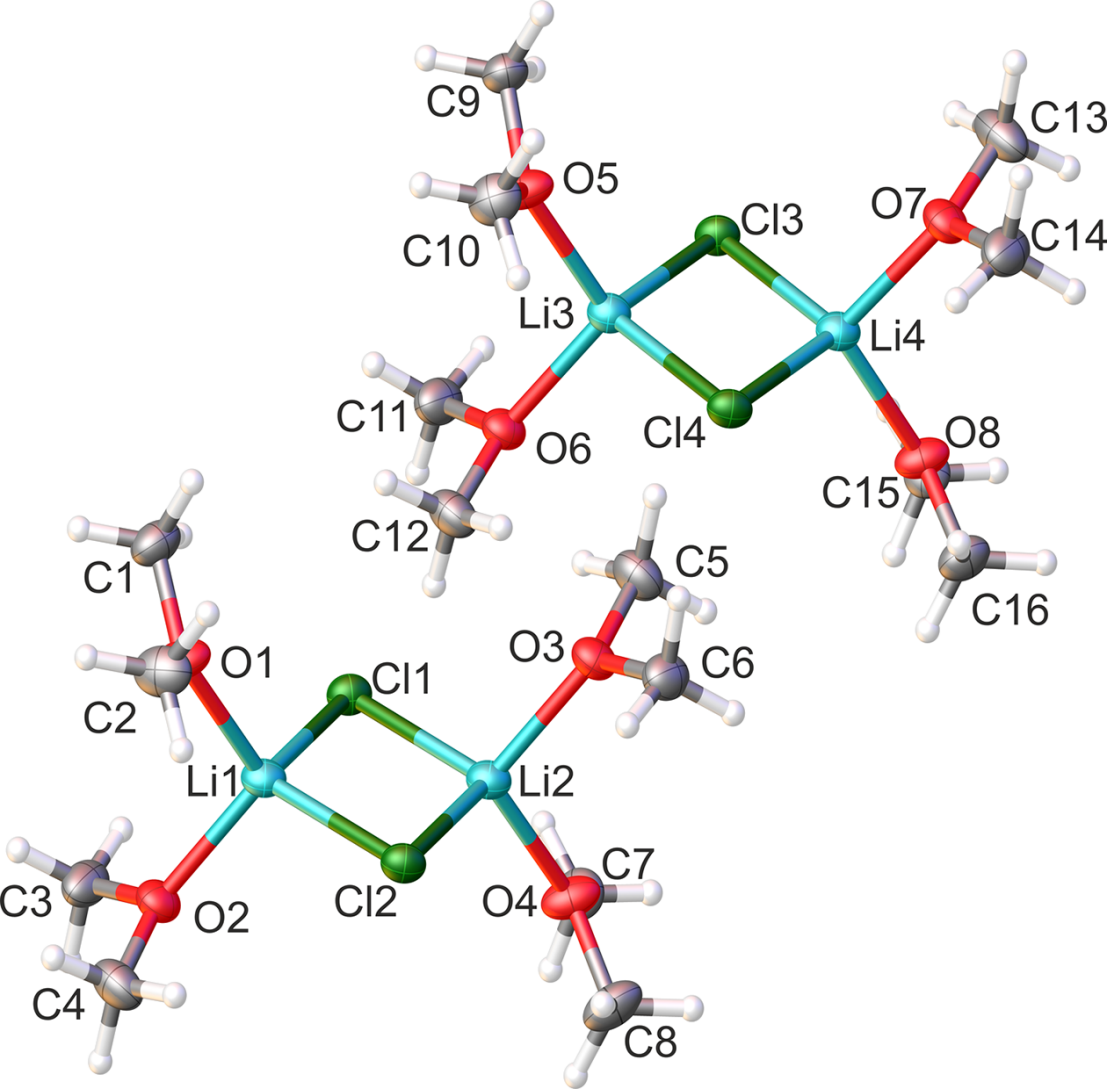
The mol­ecular structure of **1**, showing 50% probability displacement ellipsoids.

The lithium chloride dimethyl ether com­plex (**1**) crystallizes with dimethyl ether as a ligand at 193 K in the monoclinic space group *P*2_1_/*n*. The unit cell contains two symmetry-independent lithium chloride dimers, both found in general positions (*Z* = 8; *Z*′ = 2). Each lithium ion is bonded with two dimethyl ether mol­ecules and two chloride ions that bridge the lithium centres. The mol­ecular structure of **1** is shown in Fig. 1 ▸, and selected bond angles and bond lengths are shown in Table 1 ▸.

**Figure 2:**
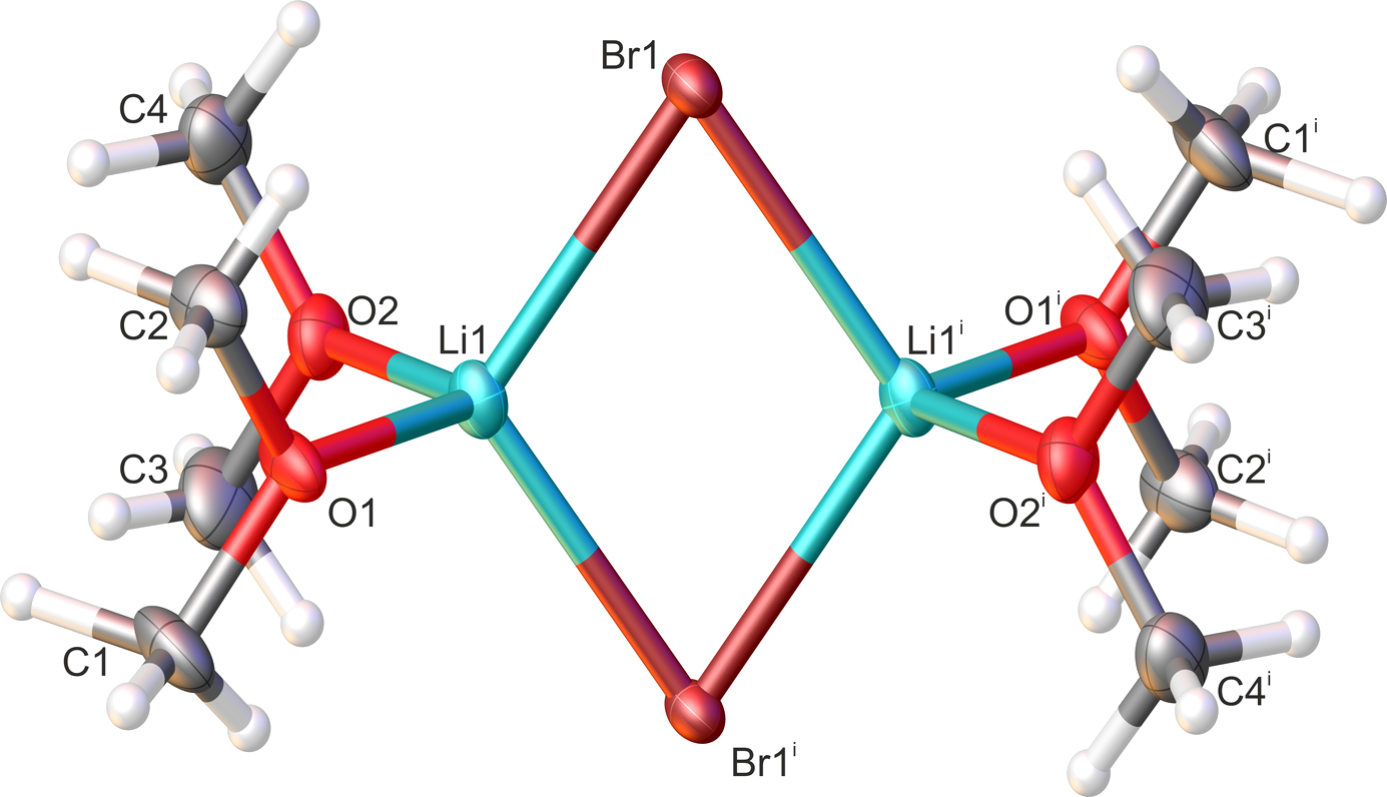
The mol­ecular structure of **2**, showing 50% probability displacement ellipsoids. The com­plete dimer is generated by inversion symmetry. [Symmetry code: (i) −*x* + 1, −*y* + 1, −*z* + 1.]

The lithium bromide dimethyl ether com­plex (**2**) also crystallizes with dimethyl ether as a ligand at 193 K in the monoclinic space group *P*2_1_/*n*. The unit cell contains two lithium bromide dimers in which each lithium ion is coordinated by two dimethyl ether mol­ecules and two bromide ions. The asymmetric unit com­prises half of a dimer, which resides across a centre of inversion. The mol­ecular structure of **2** is shown in Fig. 2 ▸ and selected bond angles and bond lengths are shown in Table 2 ▸.

A com­parison of the inter­atomic distances within the mol­ecules (see Table 3 ▸) shows that the average lithium–halogenide bond is approximately 0.18 Å shorter and the average Li⋯Li distance is 0.14 Å shorter in **1** (LiCl) than in **2** (LiBr). In contrast, the Li—O bonds show almost the same (Δ*d* = 0.02 Å) and the C—O bonds show the same distance. The lengths of the C—O bonds in the ethers are in good agreement with literature data from X-ray measurements (Allen *et al.*, 1987 ▸). The observed elongation of the Li—Br and Li⋯Li distances in **2** is consistent with the larger ionic radius of bromide relative to chloride.

In the structure of **1**, four distinct O—Li—O vectors are present in the asymmetric unit. These are not parallel, but differ by small angles, which explains why the chloride structure contains two independent dimer mol­ecules in the asymmetric unit, com­pared with the bromide structure (**2**), where the O—Li—O orientations are coherent, and the asymmetric unit com­prises only half of a dimer. This difference also suggests the possibility of polytypes of the chloride structure, differing in the sequence of packing.

## 3. Supra­molecular features

**Figure 3:**
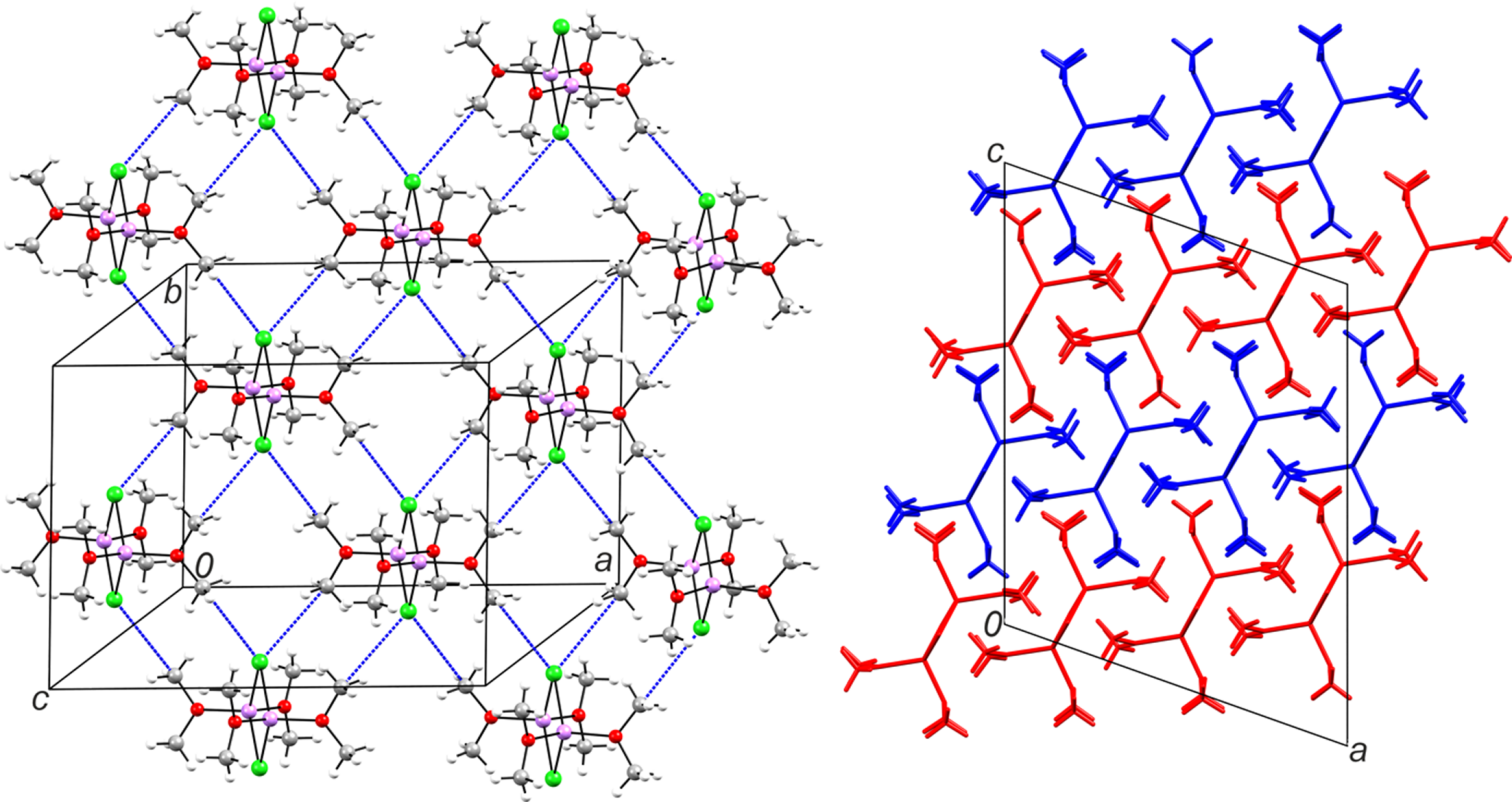
The layers in **1**. The two-dimensional 4-connected network is generated by CH_3_⋯Cl tetrel bonds (blue dashed lines) parallel to (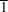
03) (left). Stacking of layers (right).

In the extended structure of **1**, the mol­ecules assemble into planar layers parallel to (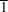
03) (see Fig. 3 ▸). Within each layer, a regular two-dimensional 4-connected network is generated by CH_3_⋯Cl tetrel bonds, in which the methyl groups attached to O1, O4, O5 and O8 are directed toward the chloride anions of neighbouring mol­ecules. The geometric parameters of these contacts, summarized in Table 4 ▸, are in the range expected for σ-hole inter­actions, with several C⋯Cl distances close to or below the van der Waals sum, and O—C⋯Cl angles ranging from close to linear to more bent arrangements. Notably, the methyl groups on O5 simultaneously engage in the strongest [C9⋯Cl4 = 3.4033 (15) Å and O5—C9⋯Cl4^v^ = 171.48 (10)°] and one of the weakest tetrel bonds [C10⋯Cl3 = 3.6179 (17) Å and O5—C10⋯Cl3^vi^ = 153.23 (12)°] [symmetry codes: (v) −*x* + 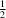, *y* + 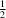, −*z* + 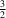; (vi) −*x* + 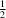, *y* − 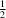, −*z* + 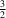] in the structure.

These tetrel bonds com­pete with C—H⋯Cl hy­dro­gen bonds, yet they act as the primary structure-determining inter­action in **1**. This behaviour aligns with the view that *sp*^3^-C-centred tetrel bonds can be structure-defining interactions (Roeleveld *et al.*, 2020 ▸) and with electronic-structure analyses showing that methyl C atoms can present an electrophilic σ-hole toward halides, giving rise to directional CH_3_⋯Hal inter­actions (Bartashevich *et al.*, 2019 ▸). More broadly, our system provides an experimental case where CH_3_-based tetrel bonds prevail over com­peting hy­dro­gen bonds, consistent with theoretical predictions that such carbon-centred tetrel bonds, though typically weak, can become structure-directing when reinforced by electronegative substituents (*e.g.* O) attached to the donor carbon atom (Scheiner, 2021 ▸). The layered morphology of **1** and lack of strong inter­layer inter­actions may suggest the possibility of polytypes, differing in the sequence of packing.

**Figure 4:**
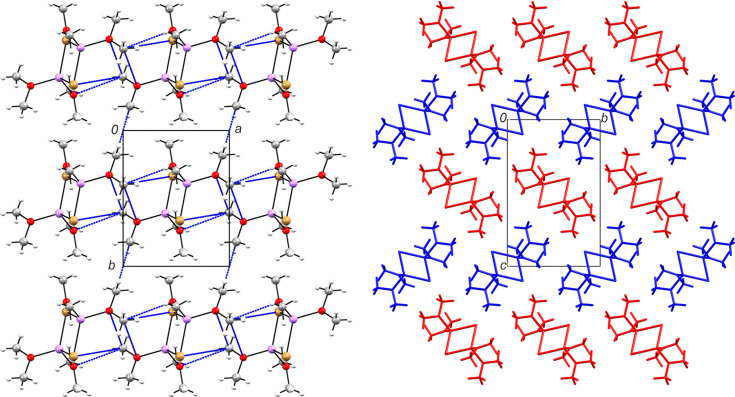
Layers in **2**. The two-dimensional network parallel to (001), viewed along [001]. CH_3_⋯CH_3_, CH_3_⋯Br and CH_3_⋯O contacts are shown as blue dashed lines (left). Stacking of *ABAB* layers (right).

In the structure of **2**, the mol­ecules form planar layers parallel to (001), in which all dimers adopt the same orientation (Fig. 4 ▸). The inter­molecular bonding in **2** is particularly weak. Unlike a markedly rich suite of tetrel inter­actions in **1**, only one such contact occurs in the present case, namely, O2—C3⋯Br1^ii^ [C3⋯Br = 3.919 (7) Å and O2—C3⋯Br1 = 161.0 (5)°; symmetry code: (ii) *x*, *y* + 1, *z*]. Another distal contact with a methyl group may reflect weak C—H⋯Br hy­dro­gen bonding [C4⋯Br1^iii^ = 4.080 (7) Å and C4—H4*B*⋯Br1^iii^ = 157 (5)°; symmetry code: (iii) −*x* + 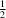, *y* + 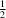, −*z* + 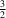]. Within each layer, the C2 methyl groups also establish distal contacts with O and Br atoms [3.480 (7) and 4.229 (6) Å, respectively], while approaching the small cage formed by LiBr_2_O_2_ tetra­hedra sharing the Br⋯Br edge. The most remarkable inter­action, however, is represented by close contacts of methyl groups, which connect the inversion-related mol­ecules in the [010] direction [C1⋯C1^iv^ = 3.350 (12) Å; symmetry code: (iv) −*x*, −*y* + 2, −*z* + 1; Fig. 4 ▸]. Such tetrel-like inter­actions are likely attractive, as was suggested by a recent study of a closely related CH_3_⋯CH_3_ di­methyl­amine dimer with *E*_tot_ = −1.7 kJ mol^−1^ (Michalczyk *et al.*, 2024 ▸).

**Figure 5:**
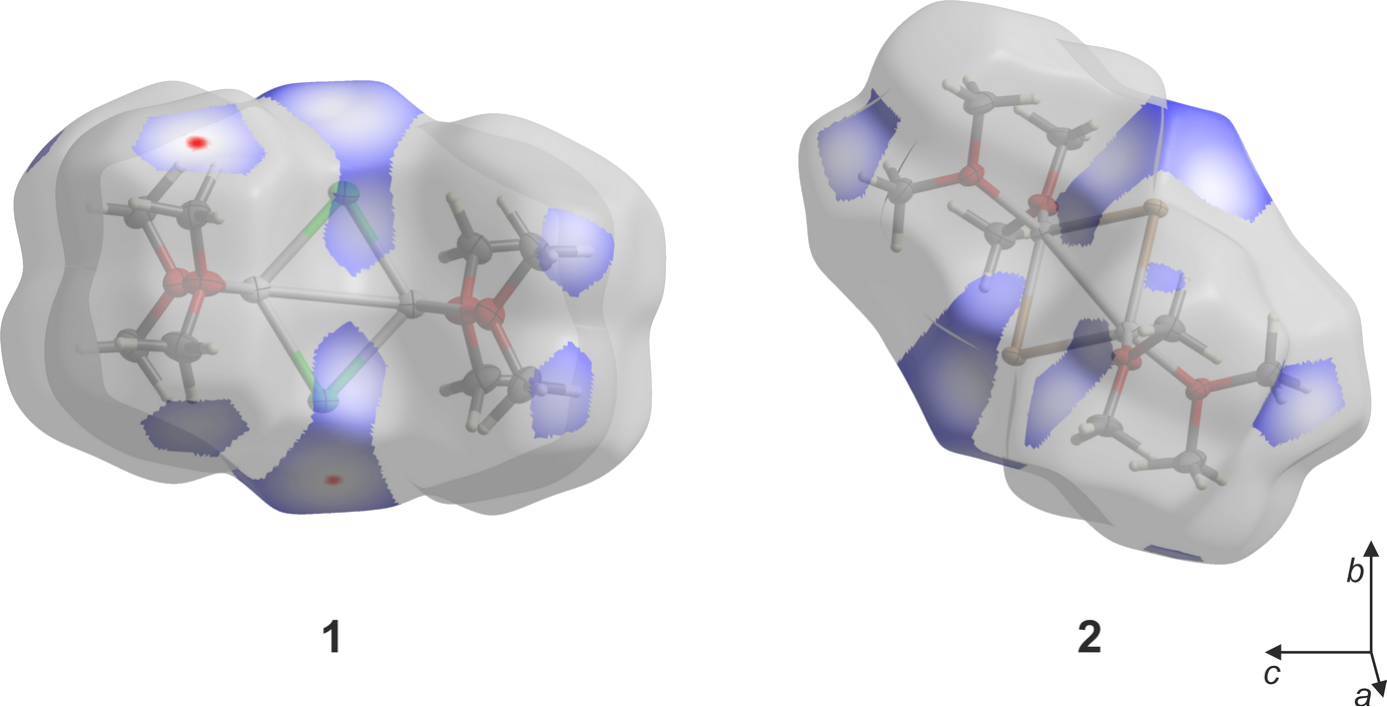
Hirshfeld surfaces of the lithium chloride (**1**) and lithium bromide (**2**) com­plexes mapped over *d*_norm_ and viewed along [100]. Coloured regions indicate halogen–hy­dro­gen contacts, while grey areas correspond to other inter­actions. Red spots highlight the closest contacts and blue areas the most distant.

To better understand the inter­molecular inter­actions, a Hirshfeld surface analysis (McKinnon *et al.*, 2007 ▸) was performed. The surfaces and corresponding fingerprint plots (Spackman & McKinnon, 2002 ▸) were calculated using *CrystalExplorer21* (Spackman *et al.*, 2021 ▸). For the lithium chloride com­plex (**1**), the Hirshfeld surface was calculated for one of the two dimers in the asymmetric unit and mapped with *d*_norm_ in the range −0.0151 to 1.1488 a.u. For the lithium bromide com­plex (**2**), the surface was mapped with *d*_norm_ in the range −0.0288 to 1.2706 a.u. Fig. 5 ▸ displays both surfaces viewed along [100]. The coloured regions on the surface correspond to halogen–hy­dro­gen inter­actions, whereas the remaining parts of the surfaces, dominated by other types of inter­actions, are shown in grey. As all H atoms in the present structures belong to methyl groups, halogen–hy­dro­gen contacts can simultaneously correspond to halogen–methyl inter­actions. Red areas represent the closest contacts, while blue areas represent the most distant ones. In com­pound **1**, the surface highlights CH_3_⋯Cl tetrel inter­actions by red spots located above a C atom, whereas CH⋯Cl contacts appear as blue regions above certain methyl H atoms. In contrast, for com­pound **2**, only the latter CH⋯Br inter­actions are observed.

**Figure 6:**
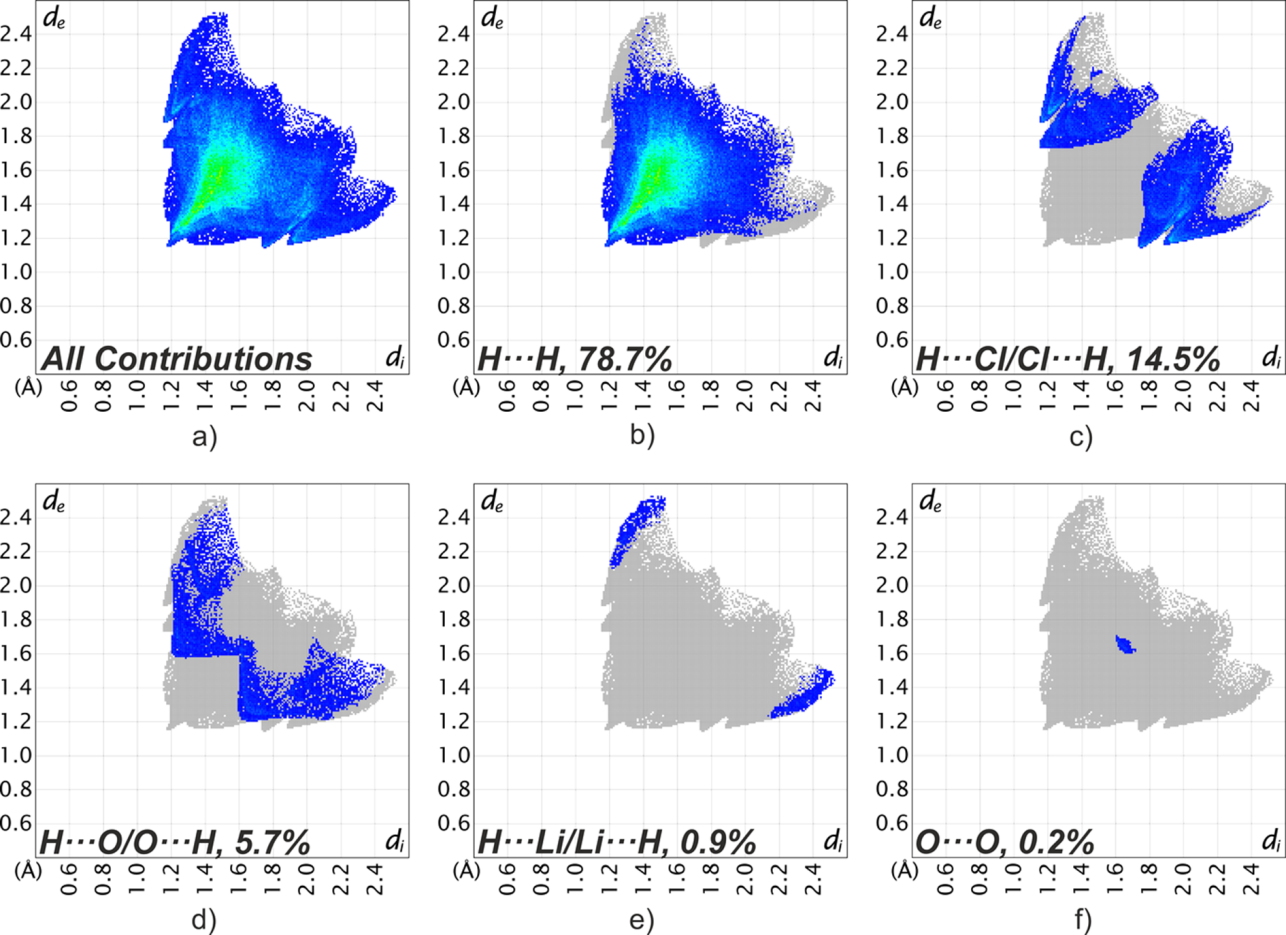
Two-dimensional fingerprint plots for **1**, showing all (*a*) and selected inter­actions (*b*)–(*f*) between atoms inside and outside the Hirshfeld surface. *d*_e_ and *d*_i_ represent the distances from a point on the Hirshfeld surface to the nearest atoms that are external or inter­nal to the surface, respectively.

The contributions of the different inter­molecular inter­actions in the lithium chloride com­plex (**1**) are summarized in the two-dimensional fingerprint plots shown in Fig. 6 ▸. These plots show that H⋯H inter­actions contribute the most to the Hirshfeld surface, at 78.7%. This is followed by H⋯Cl/Cl⋯H and H⋯O/O⋯H inter­actions, which contribute 14.5 and 5.7%, respectively. The H⋯Li/Li⋯H (0.9%) and O⋯O (0.2%) inter­actions contribute less than 1% each. The close contacts on the Hirshfeld surface (red areas, Fig. 5 ▸) show the inter­molecular tetrel bond, which contributes to the H⋯Cl/Cl⋯H inter­actions.

**Figure 7:**
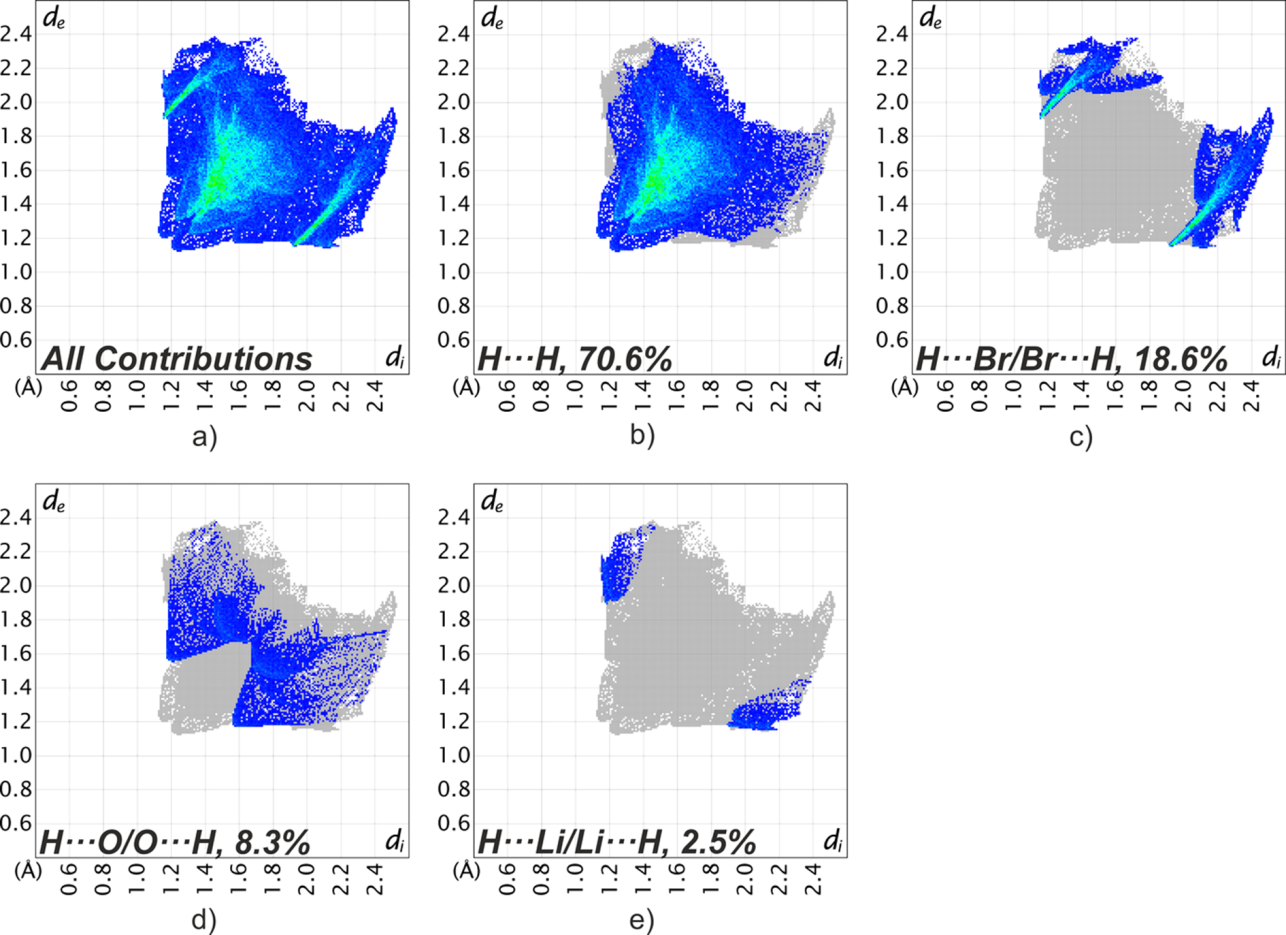
Two-dimensional fingerprint plots for **2**, showing all (*a*) and selected inter­actions (*b*)–(*e*) between atoms inside and outside of the Hirshfeld surface. *d*_e_ and *d*_i_ represent the distances from a point on the Hirshfeld surface to the nearest atoms that are external or inter­nal to the surface, respectively.

The contributions of the different inter­molecular inter­actions to the Hirshfeld surface in the lithium bromide com­plex (**2**) are summarized in Fig. 7 ▸. The fingerprint plots presented there show that the H⋯H inter­actions make the largest contribution (70.6%) to the Hirshfeld surface. The second largest contribution, at 18.6%, comes from the H⋯Br/Br⋯H inter­actions. H⋯O/O⋯H and H⋯Li/Li⋯H inter­actions account for 8.3 and 2.5% of the Hirshfeld surface, respectively. The close C1⋯C1 contact contributes to the H⋯H inter­actions on the Hirshfeld surface.

A direct com­parison of the fingerprint plots for the lithium chloride com­plex (Fig. 6 ▸) and the bromide com­plex (Fig. 7 ▸) structures (**1** and **2**) reveal clear differences in the nature of the meth­yl–halogen contacts. In the chloride com­plex, the Cl⋯H/H⋯Cl region is represented by diffuse clouds, which reflect the previously identified CH_3_⋯Cl tetrel inter­actions, rather than classical CH⋯Cl hy­dro­gen bonds. In contrast, the bromide com­plex shows two sharp spikes in the Br⋯H/H⋯Br region, indicating the presence of weak CH⋯Br hy­dro­gen bonds.

## 4. Database survey

A search in the Cambridge Structural Database (CSD; Groom *et al.*, 2016 ▸; WebCSD June 2025) for lithium chloride dimers with etheric ligands revealed four relevant entries of lithium chloride with THF ligands, which can be com­pared to com­pound **1**. The structures of lithium chloride with THF as a ligand [CSD refcodes MOZZAE (Fischer *et al.*, 2015 ▸) and VIJMAC–VIJMAC02 (Hahn & Rupprecht, 1991 ▸; Blasberg *et al.*, 2012 ▸; Knauer & Strohmann, 2020 ▸)] all feature lithium chloride dimers in which each lithium ion is bonded with two THF mol­ecules.

A structure not present in the CSD, but published separately, is that of lithium chloride bonded with diethyl ether. It features a lithium chloride tetra­mer in which each lithium ion is bonded with one ether mol­ecule (Mitzel & Lustig, 2001 ▸). When com­paring these lithium chloride aggregates with our own structure (see Table 5 ▸), the structure of non-coordinated crystaline lithium chloride was also taken into account [ICSD 26909 (Levin’sh *et al.*, 1938 ▸) and ICSD 27981 (Ott, 1923 ▸)].

The Li—Cl bond lengths of **1** are shorter than in the Et_2_O structure and lie at the lower limit found for the THF com­plexes. All ligated structures have significantly shorter Li—Cl distances com­pared to LiCl itself, which indicates more localized bonding in the ligated structures due to the reduced number of Li—Cl contacts.

The Li—O bond lengths fit well to those found in the THF-containing structures. The average Li—O distances in the Et_2_O-containing structure are smaller, suggesting stronger Li—O inter­actions resulting from the presence of only one ether mol­ecule per lithium ion, in contrast to two in the THF and Me_2_O structures.

The Li⋯Li distance of **1** is shorter than in the other structures, which may reflect the lower steric demand of dimethyl ether com­pared to THF and Et_2_O.

For lithium bromide with etheric ligands the search in the CSD identified structures with both THF and diethyl ether that can be com­pared to com­pound **2**. The THF structure (YESKEN; Vitze *et al.*, 2006 ▸) also consists of a lithium bromide dimer with each lithium ion bonded with two THF mol­ecules. The diethyl ether structures [ZIWLEW (Neumann *et al.*, 1995 ▸) and ZIWLEW01 (Spring *et al.*, 2002 ▸)] feature lithium bromide tetra­mers with each of the four lithium ions bonded with one ether mol­ecule.

When com­paring these lithium bromide derivatives with the present structure of **2** (see Table 6 ▸), the structure of lithium bromide itself was also included [ISCD 27982 (Ott, 1923 ▸), ISCD 44274 (Cortona, 1992 ▸), ISCD 52236 (Finch & Fordham, 1936 ▸), ISCD 53819 (Posnjak & Wyckoff, 1922 ▸) and ISCD 671519 (Sadigh *et al.*, 2015 ▸)].

The Li—Br bond lengths in **2** lie at the lower limit found for the THF analogs and are shorter than in the Et_2_O structures. Again, all ligated structures show shorter Li—Br distances than the structure of LiBr itself, reflecting more localized bonding due to fewer Li—Br contacts.

The Li—O bond lengths in **2** agree well with those in the THF structure, while the average distance in the Et_2_O structures are slightly shorter, indicating a stronger inter­action likely due to the single Li—O contact per lithium ion in the tetra­mers.

The Li⋯Li distance in **2** is shorter than in the other structures, which may reflect the lower steric demand of dimethyl ether com­pared to THF and Et_2_O.

## 5. Synthesis and crystallization

For the synthesis of lithium chloride com­plex **1**, chloro­butane (0.10 ml, 1.00 mmol, 1.0 equiv.) was added to 1.00 ml of diethyl ether under inert conditions. At 273 K, *tert*-butyl­lithium (1.05 ml, 1.90 *M* in *n*-pentane, 2.00 mmol, 2.0 equiv.) was added. The yellow solution was stirred for 1 h at room tem­per­a­ture. Subsequently, 20.0 ml diethyl ether was added to the colourless suspension followed by 0.50 ml of dimethyl ether at 243 K before storage at 193 K. After 1 d, product **1** was obtained as colourless blocks, which were suitable for X-ray diffraction. The crystals had to be handled with great care, as they would melt on contact with air or if they warmed above 193 K.

For the synthesis of lithium bromide com­plex **2**, di­bromo­ethane (0.09 ml, 1.00 mmol, 1.0 equiv.) was added to 1.00 ml of diethyl ether under inert conditions. At 273 K, *tert*-butyl­lithium (1.05 ml, 1.90 *M* in *n*-pentane, 2.00 mmol, 2.0 equiv.) was added. The white suspension was stirred for 1 h at room tem­per­a­ture. Subsequently, 10.0 ml diethyl ether was added to the colourless suspension and 0.50 ml dimethyl ether was added to the now clear solution at 243 K. The solution was stored at 193 K. After 1 d, product **2** was obtained as colourless blocks, which were suitable for X-ray diffraction. The crystals had to be handled with great care, as they melt on contact with air or if they warmed above 193 K.
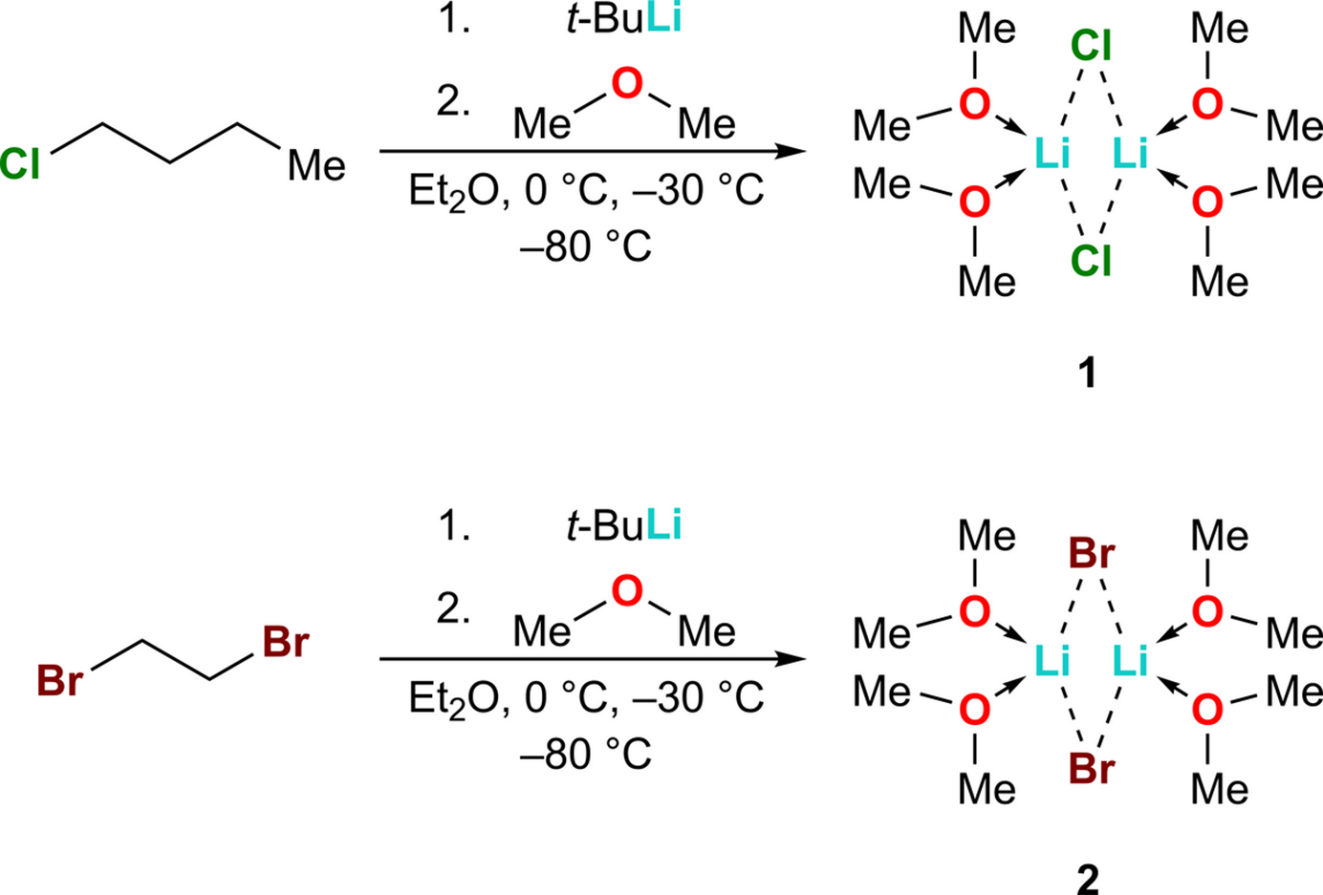


## 6. Refinement

Crystal data, data collection and structure refinement details are summarized in Table 7 ▸.

## Supplementary Material

Crystal structure: contains datablock(s) mo_b3199_0m, ag_acs_s0083_0m, New_Global_Publ_Block. DOI: 10.1107/S2056989025009119/nu2014sup1.cif

Structure factors: contains datablock(s) mo_b3199_0m. DOI: 10.1107/S2056989025009119/nu2014mo_b3199_0msup2.hkl

Structure factors: contains datablock(s) ag_acs_s0083_0m. DOI: 10.1107/S2056989025009119/nu2014ag_acs_s0083_0msup3.hkl

CCDC references: 2496191, 2496190

Additional supporting information:  crystallographic information; 3D view; checkCIF report

## Figures and Tables

**Table 1:** Selected geometric parameters (Å, °) for **1**

Li1—Cl1	2.313 (2)	Li1—O1	1.948 (3)
Li2—Cl1	2.320 (2)	Li1—O2	1.947 (2)
Li1—Cl2	2.325 (2)	Li2—O3	1.943 (2)
Li2—Cl2	2.316 (2)	Li2—O4	1.943 (2)
Li1—Li2	2.811 (3)		
			
Li1—Cl1—Li2	74.70 (7)	Cl1—Li1—Cl2	105.35 (9)
Li1—Cl2—Li2	74.55 (7)	Cl1—Li2—Cl2	105.40 (9)

**Table 2:** Selected geometric parameters (Å, °) for **2**

Li1—Br1	2.496 (9)	Li1—O1	1.912 (9)
Li1—Br1^i^	2.501 (9)	Li1—O2	1.943 (10)
Li1—Li1^i^	2.956 (17)		
			
Li1—Br1—Li1^i^	72.5 (3)	O2—Li1—Br1^i^	109.7 (4)
Br1—Li1—Br1^i^	107.5 (3)	O2—Li1—Br1	108.9 (4)
O1—Li1—Br1	115.1 (4)	O1—Li1—O2	104.0 (4)
O1—Li1—Br1^i^	111.5 (4)		

Symmetry code: (i) 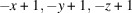.

**Table 3:** Comparison of the distances (Å) in **1** and **2** Weighted mean values were calculated for each bond type by including all unique bond lengths and using the individual uncertainties as weights.

	**1**	**2**
Li—*X*	2.3188 (7)	2.499 (7)
Li—O	1.9447 (8)	1.926 (7)
O—C	1.4195 (4)	1.423 (4)
Li⋯Li	2.814 (3)	2.956 (17)

**Table 4:** Geometric parameters (Å and °) of the tetrel bonds in **1**

O—CH3⋯Cl	C⋯Cl	O—C⋯Cl	H⋯Cl	C—H⋯Cl
O1—C1⋯Cl1^i^	3.4864 (16)	165.49 (10)	3.162 (19)–3.53 (2)	79.8 (12)–101.3 (12)
O1—C2⋯Cl2^ii^	3.5115 (17)	159.18 (13)	2.93 (2)–3.51 (3)	82.4 (16)–118.0 (14)
O4—C7⋯Cl4^iii^	3.4225 (16)	166.18 (12)	2.965 (19)–3.34 (2)	87.6 (13)–109.1 (12)
O4—C8⋯Cl3^iv^	3.5879 (19)	155.90 (13)	3.09 (2)–3.80 (2)	70.3 (13)–113.2 (14)
O5—C9⋯Cl4^v^	3.4033 (15)	171.48 (10)	3.088 (19)–3.35 (2)	84.6 (13)–101.1 (12)
O5—C10⋯Cl3^vi^	3.6179 (17)	153.23 (12)	3.55 (2)–2.97 (2)	77.4 (13)–125.2 (15)
O8—C15⋯Cl2^iii^	3.4324 (15)	169.65 (9)	3.12 (2)–3.38 (2)	86.0 (14)–100.4 (15)
O8—C16⋯Cl1^iv^	3.5822 (16)	155.04 (12)	3.59 (2)–2.974 (18)	74.6 (12)–123.8 (13)

Symmetry codes: (i) −*x* + 1, −*y* + 1, −*z* + 1; (ii) −*x* + 1, −*y*, −*z* + 1; (iii) −*x* + 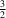, *y* + 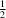, −*z* + 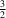; (iv) −*x* + 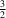, *y* − 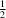, −*z* + 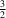; (v) −*x* + 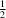, *y* + 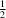,-*z* + 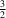; (vi) −*x* + 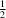, *y* − 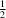, −*z* + 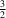.

**Table 5:** Comparison of the shortest and longest distances (Å) in [Li_2_Cl_2_(Me_2_O)_4_] (**1**) with literature-reported ligated lithium chloride structures and values for crystalline LiCl For crystalline LiCl, the values represent distances derived from multiple literature sources.

Distance	Li—Cl	Li—O	Li⋯Li
**1**	2.313 (2)–2.325 (2)	1.941 (2)–1.953 (3)	2.811 (3)–2.817 (3)
Et_2_O (Mitzel & Lustig, 2001 ▸)	2.35 (1)–2.40 (1)	1.90 (1)–1.93 (1)	3.00 (2)–3.08 (1)
THF (MOZZAE)	2.374 (5)–2.387 (5)	1.956 (5)–1.957 (5)	2.928 (10)
THF (VIJMAC)	2.308 (4)–2.341 (4)	1.937 (4)–1.956 (5)	2.896 (8)
THF (VIJMAC01)	2.320 (17)–2.368 (18)	1.922 (18)–1.962 (15)	2.93 (3)
THF (VIJMAC02)	2.313 (3)–2.344 (3)	1.941 (3)–1.959 (3)	2.903 (5)
LiCl	2.565–2.572		3.627–3.637

**Table 6:** Comparison of the shortest and longest distances (Å) in [Li_2_Cl_2_(Me_2_O)_4_] (**2**) with literature-reported ligated lithium bromide structures and averaged values for crystalline LiBr For crystalline LiBr, the values represent averaged distances derived from multiple literature sources.

Distance in Å	Li—Br	Li—O	Li⋯Li
**2**	2.496 (9)–2.501 (9)	1.912 (9)–1.943 (10)	2.956 (17)
THF (YESKEN)	2.485 (9)–2.540 (9)	1.918 (10)–1.951 (10)	3.104 (18)
Et_2_O (ZIWLEW)	2.541 (1)–2.617 (2)	1.815 (1)–1.873 (1)	3.242 (2)–3.367 (2)
Et_2_O (ZIWLEW01)	2.525 (7)–2.564 (7)	1.885 (7)–1.906 (7)	3.159 (13)–3.231 (10)
LiBr	2.740 (14)		3.875 (19)

**Table 7:** Experimental details For all structures: [Li_2_Cl_2_(C_2_H_6_O)_2_], monoclinic, *P*2_1_/*n*. Experiments were carried out at 100 K.

	**1**	**2**
Crystal data		
*M*_r_	269.05	357.97
*a*, *b*, *c* (Å)	15.1778 (11), 11.4091 (8), 19.2725 (14)	6.8459 (14), 8.7128 (18), 13.816 (3)
β (°)	109.554 (2)	94.884 (6)
*V* (Å^3^)	3144.8 (4)	821.1 (3)
*Z*	8	2
Radiation type	Mo *K*α	Ag *K*α, λ = 0.56086 Å
μ (mm^−1^)	0.41	2.64
Crystal size (mm)	0.48 × 0.37 × 0.32	0.41 × 0.18 × 0.11

Data collection		
Diffractometer	Bruker APEXII CCD	Bruker D8 VENTURE area detector
Absorption correction	–	Multi-scan (*SADABS*; Bruker, 2016 ▸)
*T*_min_, *T*_max_	–	0.321, 0.560
No. of measured, independent and observed [*I* > 2σ(*I*)] reflections	302802, 9680, 6471	13431, 1826, 1587
*R*_int_	0.049	0.058
(sin θ/λ)_max_ (Å^−1^)	0.717	0.643

Refinement		
*R*[*F*^2^ > 2σ(*F*^2^)], *wR*(*F*^2^), *S*	0.031, 0.074, 1.11	0.050, 0.126, 1.19
No. of reflections	9680	1826
No. of parameters	481	120
H-atom treatment	All H-atom parameters refined	Only H-atom coordinates refined
Δρ_max_, Δρ_min_ (e Å^−3^)	0.21, −0.18	1.12, −1.54

Computer programs: *APEX2* (Bruker, 2016 ▸), *SAINT* (Bruker, 2016 ▸), *SHELXT* (Sheldrick, 2015 ▸), *SHELXL* (Sheldrick, 2008 ▸) and *OLEX2* (Dolomanov *et al.*, 2009 ▸).

## supplementary crystallographic information

### Di-µ-chlorido-bis[bis(dimethyl ether-κO)lithium] (mo_b3199_0m) Crystal data

**Table:**

[Li_2_Cl_2_(C_2_H_6_O)_2_]	*F*(000) = 1152
*M**_r_* = 269.05	*D*_x_ = 1.137 Mg m^−^^3^
Monoclinic, *P*2_1_/*n*	Mo *K*α radiation, λ = 0.71073 Å
*a* = 15.1778 (11) Å	Cell parameters from 9994 reflections
*b* = 11.4091 (8) Å	θ = 2.7–30.6°
*c* = 19.2725 (14) Å	µ = 0.41 mm^−^^1^
β = 109.554 (2)°	*T* = 100 K
*V* = 3144.8 (4) Å^3^	Block, colourless
*Z* = 8	0.48 × 0.37 × 0.32 mm

### Di-µ-chlorido-bis[bis(dimethyl ether-κO)lithium] (mo_b3199_0m) Data collection

**Table:**

Bruker APEXII CCD diffractometer	6471 reflections with *I* > 2σ(*I*)
Radiation source: microfocus sealed X-ray tube, Incoatec Iµs	*R*_int_ = 0.049
HELIOS mirror optics monochromator	θ_max_ = 30.6°, θ_min_ = 2.1°
Detector resolution: 10.4167 pixels mm^-1^	*h* = −21→21
φ and ω scans	*k* = −16→16
302802 measured reflections	*l* = −27→27
9680 independent reflections	

### Di-µ-chlorido-bis[bis(dimethyl ether-κO)lithium] (mo_b3199_0m) Refinement

**Table:**

Refinement on *F*^2^	Primary atom site location: dual
Least-squares matrix: full	Hydrogen site location: difference Fourier map
*R*[*F*^2^ > 2σ(*F*^2^)] = 0.031	All H-atom parameters refined
*wR*(*F*^2^) = 0.074	*w* = 1/[σ^2^(*F*_o_^2^) + (0.0227*P*)^2^ + 0.7058*P*] where *P* = (*F*_o_^2^ + 2*F*_c_^2^)/3
*S* = 1.11	(Δ/σ)_max_ = 0.002
9680 reflections	Δρ_max_ = 0.21 e Å^−^^3^
481 parameters	Δρ_min_ = −0.18 e Å^−^^3^
0 restraints	

### Di-µ-chlorido-bis[bis(dimethyl ether-κO)lithium] (mo_b3199_0m) Special details

**Table:**

Geometry. All esds (except the esd in the dihedral angle between two l.s. planes) are estimated using the full covariance matrix. The cell esds are taken into account individually in the estimation of esds in distances, angles and torsion angles; correlations between esds in cell parameters are only used when they are defined by crystal symmetry. An approximate (isotropic) treatment of cell esds is used for estimating esds involving l.s. planes.

### Di-µ-chlorido-bis[bis(dimethyl ether-κO)lithium] (mo_b3199_0m) Fractional atomic coordinates and isotropic or equivalent isotropic displacement parameters (Å^2^)

**Table:**

	*x*	*y*	*z*	*U*_iso_*/*U*_eq_	
Cl1	0.68353 (2)	0.42118 (2)	0.55253 (2)	0.02543 (6)	
Cl2	0.69161 (2)	0.09949 (2)	0.57241 (2)	0.02483 (6)	
O1	0.50569 (7)	0.23978 (8)	0.43568 (6)	0.0339 (2)	
O2	0.69788 (7)	0.23407 (8)	0.41059 (5)	0.03010 (19)	
O3	0.67685 (7)	0.28287 (8)	0.71387 (5)	0.02805 (19)	
O4	0.86912 (7)	0.28167 (8)	0.68946 (6)	0.0342 (2)	
C1	0.44421 (11)	0.33636 (12)	0.42514 (9)	0.0352 (3)	
C2	0.45713 (12)	0.13151 (13)	0.41871 (10)	0.0400 (3)	
C3	0.70258 (12)	0.33073 (14)	0.36536 (9)	0.0383 (3)	
C4	0.70091 (13)	0.12458 (14)	0.37673 (9)	0.0394 (3)	
C5	0.67488 (13)	0.39161 (12)	0.75003 (9)	0.0361 (3)	
C6	0.67187 (13)	0.18530 (12)	0.75830 (9)	0.0350 (3)	
C7	0.92007 (11)	0.38788 (12)	0.69874 (9)	0.0341 (3)	
C8	0.92843 (13)	0.18285 (13)	0.71057 (11)	0.0420 (4)	
Li1	0.64013 (17)	0.24915 (16)	0.48641 (12)	0.0259 (4)	
Li2	0.73515 (16)	0.27194 (18)	0.63840 (12)	0.0254 (4)	
Cl3	0.43960 (3)	0.43718 (2)	0.81722 (2)	0.02502 (6)	
Cl4	0.43569 (3)	0.11452 (2)	0.80882 (2)	0.02426 (5)	
O5	0.25622 (7)	0.28207 (8)	0.68456 (6)	0.0323 (2)	
O6	0.44944 (7)	0.28071 (8)	0.66150 (5)	0.02776 (18)	
O7	0.42683 (7)	0.26077 (8)	0.96526 (5)	0.03015 (19)	
O8	0.61974 (7)	0.26472 (7)	0.94111 (6)	0.03080 (19)	
C9	0.19855 (11)	0.38192 (11)	0.67862 (8)	0.0310 (3)	
C10	0.20373 (12)	0.17812 (12)	0.66071 (9)	0.0359 (3)	
C11	0.45189 (13)	0.38468 (13)	0.62190 (9)	0.0373 (3)	
C12	0.45266 (12)	0.17768 (12)	0.62079 (8)	0.0350 (3)	
C13	0.42315 (13)	0.36033 (15)	1.00935 (9)	0.0430 (4)	
C14	0.42302 (13)	0.15338 (15)	1.00121 (9)	0.0411 (3)	
C15	0.68096 (10)	0.36074 (11)	0.94692 (8)	0.0301 (3)	
C16	0.66836 (11)	0.15741 (12)	0.96238 (9)	0.0352 (3)	
Li3	0.39026 (16)	0.28051 (18)	0.73655 (12)	0.0255 (4)	
Li4	0.48498 (16)	0.27073 (18)	0.88955 (12)	0.0255 (4)	
H6A	0.6656 (14)	0.1165 (16)	0.7286 (10)	0.040 (5)*	
H6B	0.7253 (18)	0.1817 (18)	0.8020 (12)	0.058 (6)*	
H5A	0.7334 (15)	0.3994 (17)	0.7940 (11)	0.050 (5)*	
H12A	0.4497 (13)	0.1103 (15)	0.6503 (9)	0.034 (4)*	
H12B	0.4000 (15)	0.1733 (15)	0.5769 (10)	0.042 (5)*	
H15A	0.7275 (14)	0.3428 (15)	0.9213 (10)	0.042 (5)*	
H11A	0.3998 (15)	0.3903 (16)	0.5799 (10)	0.046 (5)*	
H1A	0.4001 (16)	0.3241 (16)	0.4512 (10)	0.050 (5)*	
H3A	0.7027 (13)	0.3989 (15)	0.3934 (9)	0.033 (4)*	
H3B	0.6498 (14)	0.3314 (15)	0.3214 (10)	0.040 (5)*	
H5B	0.6162 (16)	0.3949 (16)	0.7642 (11)	0.052 (5)*	
H5C	0.6750 (15)	0.4554 (16)	0.7159 (10)	0.047 (5)*	
H10A	0.2474 (16)	0.1142 (18)	0.6713 (11)	0.055 (6)*	
H6C	0.6126 (15)	0.1890 (15)	0.7700 (10)	0.042 (5)*	
H10B	0.1665 (15)	0.1819 (16)	0.6118 (11)	0.047 (5)*	
H7A	0.9705 (15)	0.3834 (16)	0.6765 (10)	0.046 (5)*	
H9A	0.2368 (15)	0.4470 (17)	0.7000 (10)	0.051 (5)*	
H16A	0.6262 (15)	0.1000 (18)	0.9575 (11)	0.053 (5)*	
H4A	0.7015 (15)	0.0635 (17)	0.4126 (11)	0.051 (5)*	
H10C	0.1609 (16)	0.1617 (17)	0.6876 (11)	0.051 (5)*	
H4B	0.7557 (13)	0.1175 (15)	0.3620 (9)	0.037 (4)*	
H14A	0.4161 (16)	0.0902 (18)	0.9645 (11)	0.059 (6)*	
H4C	0.6506 (16)	0.1162 (17)	0.3341 (11)	0.051 (5)*	
H13A	0.4767 (17)	0.3637 (18)	1.0532 (12)	0.059 (6)*	
H2A	0.4182 (16)	0.1173 (17)	0.4504 (11)	0.054 (6)*	
H11B	0.5046 (13)	0.3864 (14)	0.6052 (9)	0.036 (4)*	
H16B	0.7073 (14)	0.1414 (15)	0.9346 (9)	0.039 (4)*	
H3C	0.7578 (14)	0.3238 (15)	0.3515 (10)	0.040 (5)*	
H14B	0.4777 (16)	0.1447 (18)	1.0453 (11)	0.057 (6)*	
H15B	0.6457 (16)	0.4303 (18)	0.9253 (11)	0.059 (6)*	
H9B	0.1561 (15)	0.3702 (16)	0.7051 (10)	0.045 (5)*	
H12C	0.5066 (14)	0.1799 (14)	0.6057 (9)	0.035 (4)*	
H9C	0.1640 (14)	0.3967 (16)	0.6269 (10)	0.046 (5)*	
H11C	0.4560 (16)	0.4525 (19)	0.6556 (11)	0.061 (6)*	
H7B	0.8784 (16)	0.4524 (18)	0.6792 (11)	0.058 (6)*	
H15C	0.7120 (14)	0.3787 (16)	0.9943 (10)	0.044 (5)*	
H8A	0.9724 (17)	0.1809 (17)	0.6831 (11)	0.055 (6)*	
H13B	0.4212 (16)	0.4311 (19)	0.9814 (12)	0.062 (6)*	
H7C	0.9520 (14)	0.4028 (15)	0.7472 (10)	0.044 (5)*	
H16C	0.7039 (15)	0.1624 (16)	1.0167 (11)	0.051 (5)*	
H1B	0.4799 (13)	0.4050 (15)	0.4443 (9)	0.039 (4)*	
H2B	0.5007 (17)	0.0703 (19)	0.4264 (11)	0.064 (6)*	
H13C	0.3635 (15)	0.3540 (16)	1.0208 (10)	0.048 (5)*	
H14C	0.3653 (14)	0.1516 (15)	1.0141 (10)	0.043 (5)*	
H8B	0.9620 (17)	0.1880 (17)	0.7638 (12)	0.056 (6)*	
H2C	0.4171 (14)	0.1314 (16)	0.3680 (11)	0.048 (5)*	
H1C	0.4072 (14)	0.3427 (16)	0.3744 (10)	0.046 (5)*	
H8C	0.8926 (16)	0.1183 (18)	0.7018 (11)	0.055 (6)*	

### Di-µ-chlorido-bis[bis(dimethyl ether-κO)lithium] (mo_b3199_0m) Atomic displacement parameters (Å^2^)

**Table:**

	*U*^11^	*U*^22^	*U*^33^	*U*^12^	*U*^13^	*U*^23^
Cl1	0.02494 (14)	0.01972 (10)	0.03317 (14)	−0.00018 (9)	0.01175 (11)	0.00162 (9)
Cl2	0.02517 (14)	0.01918 (10)	0.03273 (14)	−0.00032 (9)	0.01312 (11)	−0.00063 (9)
O1	0.0187 (5)	0.0288 (4)	0.0526 (6)	−0.0005 (3)	0.0100 (4)	−0.0017 (4)
O2	0.0298 (5)	0.0349 (4)	0.0314 (4)	0.0015 (4)	0.0179 (4)	0.0014 (3)
O3	0.0297 (5)	0.0291 (4)	0.0309 (4)	−0.0010 (3)	0.0174 (4)	−0.0012 (3)
O4	0.0196 (5)	0.0241 (4)	0.0573 (6)	−0.0016 (3)	0.0107 (4)	0.0015 (4)
C1	0.0265 (7)	0.0359 (6)	0.0419 (7)	0.0056 (5)	0.0097 (6)	0.0091 (5)
C2	0.0303 (8)	0.0349 (6)	0.0529 (9)	−0.0080 (5)	0.0115 (6)	−0.0141 (6)
C3	0.0300 (8)	0.0502 (8)	0.0391 (7)	0.0082 (6)	0.0176 (6)	0.0161 (6)
C4	0.0379 (9)	0.0467 (7)	0.0390 (7)	−0.0061 (6)	0.0200 (7)	−0.0136 (6)
C5	0.0386 (9)	0.0360 (6)	0.0382 (7)	−0.0055 (6)	0.0188 (7)	−0.0103 (5)
C6	0.0345 (9)	0.0386 (6)	0.0366 (7)	0.0048 (5)	0.0183 (6)	0.0093 (5)
C7	0.0295 (7)	0.0304 (6)	0.0400 (7)	−0.0078 (5)	0.0086 (6)	−0.0048 (5)
C8	0.0289 (8)	0.0329 (6)	0.0591 (10)	0.0060 (5)	0.0078 (7)	0.0110 (6)
Li1	0.0211 (11)	0.0270 (9)	0.0328 (10)	−0.0002 (7)	0.0131 (8)	−0.0003 (7)
Li2	0.0222 (11)	0.0244 (8)	0.0324 (10)	−0.0007 (8)	0.0130 (8)	−0.0015 (7)
Cl3	0.02503 (11)	0.01779 (9)	0.03315 (12)	−0.00034 (10)	0.01093 (9)	−0.00178 (9)
Cl4	0.02525 (11)	0.01751 (9)	0.03301 (12)	0.00010 (10)	0.01371 (9)	−0.00004 (9)
O5	0.0192 (5)	0.0230 (4)	0.0528 (6)	0.0000 (3)	0.0095 (4)	−0.0035 (4)
O6	0.0283 (5)	0.0296 (4)	0.0305 (4)	0.0014 (3)	0.0167 (4)	0.0012 (3)
O7	0.0283 (5)	0.0367 (4)	0.0307 (4)	−0.0026 (3)	0.0170 (4)	−0.0026 (3)
O8	0.0186 (4)	0.0238 (3)	0.0492 (5)	−0.0002 (3)	0.0103 (4)	0.0021 (3)
C9	0.0282 (7)	0.0301 (5)	0.0345 (6)	0.0078 (5)	0.0102 (5)	0.0012 (4)
C10	0.0303 (8)	0.0293 (6)	0.0452 (8)	−0.0069 (5)	0.0088 (6)	−0.0052 (5)
C11	0.0360 (9)	0.0412 (7)	0.0396 (7)	0.0079 (6)	0.0194 (7)	0.0130 (6)
C12	0.0326 (8)	0.0409 (7)	0.0356 (7)	−0.0055 (5)	0.0169 (6)	−0.0116 (5)
C13	0.0356 (9)	0.0570 (9)	0.0428 (8)	−0.0120 (7)	0.0217 (7)	−0.0215 (7)
C14	0.0356 (8)	0.0534 (8)	0.0399 (8)	0.0074 (7)	0.0200 (6)	0.0154 (6)
C15	0.0238 (6)	0.0309 (5)	0.0349 (6)	−0.0067 (5)	0.0091 (5)	−0.0020 (5)
C16	0.0297 (7)	0.0298 (6)	0.0446 (8)	0.0061 (5)	0.0105 (6)	0.0070 (5)
Li3	0.0232 (12)	0.0248 (8)	0.0307 (10)	−0.0004 (8)	0.0120 (9)	−0.0010 (7)
Li4	0.0228 (11)	0.0255 (8)	0.0316 (10)	−0.0001 (8)	0.0134 (8)	−0.0001 (7)

### Di-µ-chlorido-bis[bis(dimethyl ether-κO)lithium] (mo_b3199_0m) Geometric parameters (Å, º)

**Table:**

Li1—Cl1	2.313 (2)	Cl3—Li3	2.322 (2)
Li2—Cl1	2.320 (2)	Cl3—Li4	2.320 (2)
Li1—Cl2	2.325 (2)	Cl4—Li3	2.315 (2)
Li2—Cl2	2.316 (2)	Cl4—Li4	2.319 (2)
Li1—Li2	2.811 (3)	O5—C9	1.4177 (15)
Li1—O1	1.948 (3)	O5—C10	1.4168 (16)
Li1—O2	1.947 (2)	O5—Li3	1.944 (2)
Li2—O3	1.943 (2)	O6—C11	1.4177 (16)
Li2—O4	1.943 (2)	O6—C12	1.4232 (15)
O1—C1	1.4140 (17)	O6—Li3	1.941 (2)
O1—C2	1.4194 (16)	O7—C13	1.4309 (17)
O2—C3	1.4222 (17)	O7—C14	1.4182 (18)
O2—C4	1.4173 (17)	O7—Li4	1.945 (2)
O3—C5	1.4281 (16)	O8—C15	1.4165 (15)
O3—C6	1.4217 (16)	O8—C16	1.4175 (16)
O4—C7	1.4162 (16)	O8—Li4	1.953 (3)
O4—C8	1.4149 (17)	C9—H9A	0.95 (2)
C1—H1A	0.97 (2)	C9—H9B	0.96 (2)
C1—H1B	0.954 (18)	C9—H9C	0.973 (19)
C1—H1C	0.954 (19)	C10—H10A	0.96 (2)
C2—H2A	1.00 (2)	C10—H10B	0.92 (2)
C2—H2B	0.94 (2)	C10—H10C	0.98 (2)
C2—H2C	0.96 (2)	C11—H11A	0.92 (2)
C3—H3A	0.947 (17)	C11—H11B	0.958 (19)
C3—H3B	0.952 (19)	C11—H11C	1.00 (2)
C3—H3C	0.96 (2)	C12—H12A	0.966 (17)
C4—H4A	0.98 (2)	C12—H12B	0.950 (19)
C4—H4B	0.967 (19)	C12—H12C	0.96 (2)
C4—H4C	0.92 (2)	C13—H13A	0.96 (2)
C5—H5A	1.01 (2)	C13—H13B	0.97 (2)
C5—H5B	1.02 (2)	C13—H13C	1.00 (2)
C5—H5C	0.981 (19)	C14—H14A	0.99 (2)
C6—H6A	0.958 (18)	C14—H14B	0.97 (2)
C6—H6B	0.96 (2)	C14—H14C	0.99 (2)
C6—H6C	1.00 (2)	C15—H15A	1.01 (2)
C7—H7A	0.99 (2)	C15—H15B	0.97 (2)
C7—H7B	0.96 (2)	C15—H15C	0.899 (19)
C7—H7C	0.912 (19)	C16—H16A	0.90 (2)
C8—H8A	0.98 (2)	C16—H16B	0.938 (19)
C8—H8B	0.98 (2)	C16—H16C	1.01 (2)
C8—H8C	0.90 (2)	Li3—Li4	2.817 (3)
			
Li1—Cl1—Li2	74.70 (7)	Li4—Cl3—Li3	74.73 (6)
Li1—Cl2—L2	74.55 (7)	Li3—Cl4—Li4	74.88 (6)
C1—O1—C2	112.22 (12)	C9—O5—Li3	124.08 (11)
C1—O1—Li1	124.01 (10)	C10—O5—C9	112.42 (11)
C2—O1—Li1	122.64 (11)	C10—O5—Li3	122.51 (11)
C3—O2—Li1	121.27 (11)	C11—O6—C12	112.48 (11)
C4—O2—C3	112.65 (11)	C11—O6—Li3	120.28 (11)
C4—O2—Li1	121.04 (11)	C12—O6—Li3	121.31 (11)
C5—O3—Li2	120.68 (10)	C13—O7—Li4	121.13 (11)
C6—O3—C5	111.86 (10)	C14—O7—C13	112.30 (12)
C6—O3—Li2	122.00 (10)	C14—O7—Li4	121.33 (11)
C7—O4—Li2	123.19 (11)	C15—O8—C16	112.39 (11)
C8—O4—C7	112.18 (12)	C15—O8—Li4	124.10 (10)
C8—O4—Li2	123.87 (11)	C16—O8—Li4	122.18 (11)
O1—C1—H1A	110.4 (11)	O5—C9—H9A	108.8 (12)
O1—C1—H1B	108.8 (11)	O5—C9—H9B	110.6 (11)
O1—C1—H1C	109.9 (12)	O5—C9—H9C	109.2 (11)
H1A—C1—H1B	108.6 (15)	H9A—C9—H9B	107.5 (16)
H1A—C1—H1C	105.6 (17)	H9A—C9—H9C	110.5 (15)
H1B—C1—H1C	113.5 (15)	H9B—C9—H9C	110.1 (16)
O1—C2—H2A	111.7 (12)	O5—C10—H10A	107.1 (13)
O1—C2—H2B	109.1 (14)	O5—C10—H10B	112.1 (12)
O1—C2—H2C	109.7 (11)	O5—C10—H10C	113.0 (12)
H2A—C2—H2B	108.3 (17)	H10A—C10—H10B	113.3 (16)
H2A—C2—H2C	108.8 (17)	H10A—C10—H10C	106.0 (16)
H2B—C2—H2C	109.3 (17)	H10B—C10—H10C	105.2 (18)
O2—C3—H3A	106.2 (10)	O6—C11—H11A	111.1 (12)
O2—C3—H3B	110.6 (11)	O6—C11—H11B	112.0 (10)
O2—C3—H3C	109.4 (10)	O6—C11—H11C	107.7 (12)
H3A—C3—H3B	110.0 (15)	H11A—C11—H11B	105.7 (15)
H3A—C3—H3C	112.9 (15)	H11A—C11—H11C	111.3 (17)
H3B—C3—H3C	107.7 (15)	H11B—C11—H11C	109.0 (16)
O2—C4—H4A	107.2 (12)	O6—C12—H12A	108.4 (10)
O2—C4—H4B	111.9 (10)	O6—C12—H12B	111.1 (11)
O2—C4—H4C	110.9 (13)	O6—C12—H12C	109.7 (10)
H4A—C4—H4B	110.0 (15)	H12A—C12—H12B	106.9 (15)
H4A—C4—H4C	111.4 (17)	H12A—C12—H12C	114.4 (14)
H4B—C4—H4C	105.5 (16)	H12B—C12—H12C	106.3 (15)
O3—C5—H5A	109.3 (11)	O7—C13—H13A	111.4 (13)
O3—C5—H5B	109.1 (11)	O7—C13—H13B	109.5 (13)
O3—C5—H5C	108.1 (11)	O7—C13—H13C	106.9 (11)
H5A—C5—H5B	112.2 (15)	H13A—C13—H13B	108.5 (18)
H5A—C5—H5C	107.5 (16)	H13A—C13—H13C	111.7 (16)
H5B—C5—H5C	110.6 (16)	H13B—C13—H13C	108.7 (17)
O3—C6—H6A	107.2 (11)	O7—C14—H14A	106.9 (12)
O3—C6—H6B	111.4 (13)	O7—C14—H14B	110.6 (13)
O3—C6—H6C	109.6 (10)	O7—C14—H14C	109.0 (10)
H6A—C6—H6B	112.2 (16)	H14A—C14—H14B	113.8 (17)
H6A—C6—H6C	104.7 (15)	H14A—C14—H14C	105.9 (16)
H6B—C6—H6C	111.5 (17)	H14B—C14—H14C	110.4 (16)
O4—C7—H7A	111.6 (11)	O8—C15—H15A	110.7 (10)
O4—C7—H7B	110.2 (13)	O8—C15—H15B	110.3 (13)
O4—C7—H7C	111.7 (12)	O8—C15—H15C	111.3 (12)
H7A—C7—H7B	111.7 (16)	H15A—C15—H15B	109.3 (16)
H7A—C7—H7C	103.2 (16)	H15A—C15—H15C	109.2 (16)
H7B—C7—H7C	108.2 (16)	H15B—C15—H15C	106.0 (16)
O4—C8—H8A	110.2 (12)	O8—C16—H16A	108.4 (14)
O4—C8—H8B	108.1 (12)	O8—C16—H16B	111.7 (11)
O4—C8—H8C	108.2 (14)	O8—C16—H16C	107.3 (11)
H8A—C8—H8B	110.8 (19)	H16A—C16—H16B	111.2 (17)
H8A—C8—H8C	111.0 (17)	H16A—C16—H16C	105.4 (16)
H8B—C8—H8C	108.4 (17)	H16B—C16—H16C	112.6 (16)
Cl1—Li1—Cl2	105.35 (9)	Cl3—Li3—Li4	52.60 (6)
Cl1—Li1—Li2	52.77 (6)	Cl4—Li3—Cl3	105.22 (8)
Cl2—Li1—Li2	52.57 (6)	Cl4—Li3—Li4	52.62 (6)
O1—Li1—Cl1	112.36 (10)	O5—Li3—Cl3	112.67 (10)
O1—Li1—Cl2	111.29 (10)	O5—Li3—Cl4	111.55 (10)
O1—Li1—Li2	127.88 (10)	O5—Li3—Li4	128.27 (10)
O2—Li1—Cl1	111.83 (10)	O6—Li3—Cl3	111.83 (10)
O2—Li1—Cl2	109.99 (10)	O6—Li3—Cl4	109.29 (10)
O2—Li1—O1	106.11 (11)	O6—Li3—O5	106.34 (11)
O2—Li1—Li2	125.98 (11)	O6—Li3—Li4	125.36 (10)
Cl1—Li2—Li1	52.52 (6)	Cl3—Li4—Li3	52.68 (6)
Cl1—Li2—Cl2	105.40 (9)	Cl4—Li4—Cl3	105.17 (9)
Cl2—Li2—Li1	52.88 (6)	Cl4—Li4—Li3	52.49 (6)
O3—Li2—Cl1	110.93 (10)	O7—Li4—Cl3	112.37 (10)
O3—Li2—Cl2	110.36 (10)	O7—Li4—Cl4	109.45 (10)
O3—Li2—O4	106.19 (11)	O7—Li4—O8	106.09 (11)
O3—Li2—Li1	125.64 (10)	O7—Li4—Li3	125.93 (10)
O4—Li2—Cl1	111.44 (10)	O8—Li4—Cl3	112.59 (10)
O4—Li2—Cl2	112.61 (10)	O8—Li4—Cl4	111.23 (10)
O4—Li2—Li1	128.17 (10)	O8—Li4—Li3	127.91 (10)

### Di-µ-bromido-bis[bis(dimethyl ether-κO)lithium] (ag_acs_s0083_0m) Crystal data

**Table:**

[Li_2_Cl_2_(C_2_H_6_O)_2_]	*F*(000) = 360
*M**_r_* = 357.97	*D*_x_ = 1.448 Mg m^−^^3^
Monoclinic, *P*2_1_/*n*	Ag *K*α radiation, λ = 0.56086 Å
*a* = 6.8459 (14) Å	Cell parameters from 96 reflections
*b* = 8.7128 (18) Å	θ = 3.7–16.5°
*c* = 13.816 (3) Å	µ = 2.64 mm^−^^1^
β = 94.884 (6)°	*T* = 100 K
*V* = 821.1 (3) Å^3^	Block, colourless
*Z* = 2	0.41 × 0.18 × 0.11 mm

### Di-µ-bromido-bis[bis(dimethyl ether-κO)lithium] (ag_acs_s0083_0m) Data collection

**Table:**

Bruker D8 VENTURE area detector diffractometer	1826 independent reflections
Radiation source: microfocus sealed X-ray tube, Incoatec Iµs	1587 reflections with *I* > 2σ(*I*)
HELIOS mirror optics monochromator	*R*_int_ = 0.058
Detector resolution: 10.4167 pixels mm^-1^	θ_max_ = 21.1°, θ_min_ = 2.2°
ω and φ scans	*h* = −8→8
Absorption correction: multi-scan (SADABS; Bruker, 2016)	*k* = −10→11
*T*_min_ = 0.321, *T*_max_ = 0.560	*l* = −17→17
13431 measured reflections	

### Di-µ-bromido-bis[bis(dimethyl ether-κO)lithium] (ag_acs_s0083_0m) Refinement

**Table:**

Refinement on *F*^2^	Primary atom site location: dual
Least-squares matrix: full	Hydrogen site location: difference Fourier map
*R*[*F*^2^ > 2σ(*F*^2^)] = 0.050	Only H-atom coordinates refined
*wR*(*F*^2^) = 0.126	*w* = 1/[σ^2^(*F*_o_^2^) + (0.0506*P*)^2^ + 3.8222*P*] where *P* = (*F*_o_^2^ + 2*F*_c_^2^)/3
*S* = 1.19	(Δ/σ)_max_ = 0.001
1826 reflections	Δρ_max_ = 1.12 e Å^−^^3^
120 parameters	Δρ_min_ = −1.54 e Å^−^^3^
0 restraints	

### Di-µ-bromido-bis[bis(dimethyl ether-κO)lithium] (ag_acs_s0083_0m) Special details

**Table:**

Geometry. All esds (except the esd in the dihedral angle between two l.s. planes) are estimated using the full covariance matrix. The cell esds are taken into account individually in the estimation of esds in distances, angles and torsion angles; correlations between esds in cell parameters are only used when they are defined by crystal symmetry. An approximate (isotropic) treatment of cell esds is used for estimating esds involving l.s. planes.

### Di-µ-bromido-bis[bis(dimethyl ether-κO)lithium] (ag_acs_s0083_0m) Fractional atomic coordinates and isotropic or equivalent isotropic displacement parameters (Å^2^)

**Table:**

	*x*	*y*	*z*	*U*_iso_*/*U*_eq_	
Br1	0.46638 (7)	0.33559 (6)	0.59974 (3)	0.01973 (18)	
O1	0.1307 (5)	0.6713 (4)	0.5585 (3)	0.0256 (8)	
O2	0.5328 (6)	0.7382 (4)	0.6601 (3)	0.0254 (8)	
C1	0.0655 (10)	0.8190 (7)	0.5279 (5)	0.0354 (14)	
C2	−0.0030 (9)	0.6028 (8)	0.6194 (5)	0.0298 (13)	
C3	0.5656 (12)	0.8980 (8)	0.6473 (6)	0.0398 (15)	
C4	0.5048 (10)	0.7023 (8)	0.7588 (4)	0.0319 (13)	
Li1	0.3993 (12)	0.6109 (10)	0.5596 (6)	0.0201 (17)	
H3A	0.453 (8)	0.940 (6)	0.661 (4)	0.009 (13)*	
H2A	−0.005 (10)	0.665 (7)	0.681 (5)	0.029 (17)*	
H3B	0.580 (9)	0.907 (7)	0.578 (4)	0.022 (15)*	
H3C	0.680 (10)	0.936 (8)	0.689 (5)	0.030 (17)*	
H4A	0.603 (11)	0.729 (9)	0.802 (5)	0.04 (2)*	
H4B	0.381 (11)	0.755 (9)	0.775 (5)	0.04 (2)*	
H1A	0.043 (10)	0.889 (8)	0.596 (5)	0.035 (18)*	
H2B	−0.130 (11)	0.608 (8)	0.587 (5)	0.033 (18)*	
H2C	0.040 (12)	0.506 (10)	0.638 (6)	0.06 (2)*	
H1B	−0.077 (14)	0.826 (10)	0.501 (7)	0.07 (3)*	
H4C	0.489 (10)	0.583 (9)	0.765 (5)	0.037 (19)*	
H1C	0.174 (13)	0.846 (9)	0.493 (6)	0.056*	

### Di-µ-bromido-bis[bis(dimethyl ether-κO)lithium] (ag_acs_s0083_0m) Atomic displacement parameters (Å^2^)

**Table:**

	*U*^11^	*U*^22^	*U*^33^	*U*^12^	*U*^13^	*U*^23^
Br1	0.0197 (3)	0.0152 (3)	0.0248 (3)	0.00269 (19)	0.00490 (17)	0.0034 (2)
O1	0.0196 (18)	0.0203 (19)	0.038 (2)	0.0065 (15)	0.0093 (15)	0.0073 (17)
O2	0.028 (2)	0.0210 (19)	0.0281 (19)	−0.0019 (16)	0.0051 (15)	−0.0064 (16)
C1	0.031 (3)	0.021 (3)	0.054 (4)	0.009 (3)	0.007 (3)	0.009 (3)
C2	0.020 (3)	0.030 (3)	0.040 (3)	0.003 (2)	0.007 (2)	0.007 (3)
C3	0.051 (4)	0.023 (3)	0.046 (4)	−0.009 (3)	0.011 (3)	−0.008 (3)
C4	0.036 (3)	0.029 (3)	0.029 (3)	0.003 (3)	−0.003 (3)	−0.006 (2)
Li1	0.014 (4)	0.020 (4)	0.026 (4)	0.002 (3)	0.000 (3)	−0.003 (3)

### Di-µ-bromido-bis[bis(dimethyl ether-κO)lithium] (ag_acs_s0083_0m) Geometric parameters (Å, º)

**Table:**

Li1—Br1	2.496 (9)	C1—H1C	0.95 (9)
Li1—Br1^i^	2.501 (9)	C2—H2A	1.01 (7)
Li1—Li1^i^	2.956 (17)	C2—H2B	0.94 (7)
Li1—O1	1.912 (9)	C2—H2C	0.92 (9)
Li1—O2	1.943 (10)	C3—H3A	0.89 (6)
O1—C1	1.415 (7)	C3—H3B	0.97 (6)
O1—C2	1.425 (7)	C3—H3C	0.99 (7)
O2—C3	1.424 (8)	C4—H4A	0.88 (8)
O2—C4	1.428 (7)	C4—H4B	1.01 (8)
C1—H1A	1.14 (7)	C4—H4C	1.05 (8)
C1—H1B	1.02 (9)		
			
Li1—Br1—Li1^i^	72.5 (3)	O2—C3—H3C	112 (4)
C1—O1—C2	110.8 (5)	H3A—C3—H3B	110 (5)
C1—O1—Li1	122.1 (5)	H3A—C3—H3C	113 (5)
C2—O1—Li1	123.1 (4)	H3B—C3—H3C	114 (5)
C3—O2—C4	111.6 (5)	O2—C4—H4A	115 (5)
C3—O2—Li1	122.6 (5)	O2—C4—H4B	107 (4)
C4—O2—Li1	117.7 (5)	O2—C4—H4C	108 (4)
O1—C1—H1A	107 (4)	H4A—C4—H4B	110 (6)
O1—C1—H1B	115 (5)	H4A—C4—H4C	106 (6)
O1—C1—H1C	98 (5)	H4B—C4—H4C	110 (6)
H1A—C1—H1B	94 (6)	Br1—Li1—Br1^i^	107.5 (3)
H1A—C1—H1C	117 (6)	Br1—Li1—Li1^i^	53.8 (3)
H1B—C1—H1C	125 (7)	Br1^i^—Li1—Li1^i^	53.7 (3)
O1—C2—H2A	109 (4)	O1—Li1—Br1	115.1 (4)
O1—C2—H2B	108 (4)	O1—Li1—Br1^i^	111.5 (4)
O1—C2—H2C	110 (5)	O1—Li1—Li1^i^	132.0 (5)
H2A—C2—H2B	107 (6)	O2—Li1—Br1^i^	109.7 (4)
H2A—C2—H2C	107 (6)	O2—Li1—Br1	108.9 (4)
H2B—C2—H2C	116 (7)	O2—Li1—Li1^i^	123.9 (5)
O2—C3—H3A	103 (4)	O1—Li1—O2	104.0 (4)
O2—C3—H3B	104 (4)		

Symmetry code: (i) −*x*+1, −*y*+1, −*z*+1.

### Geometric parameters (Å and °) of the tetrel bonds in 1

**Table:**

O—CH_3_···Cl	C···Cl	O—C···Cl	HA···Cl	HB···Cl	HC···Cl	C—HA···Cl	C—HB···Cl	C—HC···Cl
O1—C1···Cl1	3.4864 (16)	165.49 (10)	3.162 (19)	3.192 (19)	3.53 (2)	101.3 (12)	100.0 (13)	79.8 (12)
O1—C2···Cl2	3.5115 (17)	159.18 (13)	2.93 (2)	3.50 (2)	3.51 (3)	118.0 (14)	82.4 (16)	82.8 (13)
O4—C7···Cl4	3.4225 (16)	166.18 (12)	2.965 (19)	3.32 (2)	3.34 (2)	109.1 (12)	88.1 (15)	87.6 (13)
O4—C8···Cl3	3.5879 (19)	155.90 (13)	3.09 (2)	3.80 (2)	3.39 (2)	113.2 (14)	70.3 (13)	95.2 (17)
O5—C9···Cl4	3.4033 (15)	171.48 (10)	3.20 (2)	3.088 (19)	3.35 (2)	94.2 (14)	101.1 (12)	84.6 (13)
O5—C10···Cl3	3.6179 (17)	153.23 (12)	3.55 (2)	3.71 (2)	2.97 (2)	86.3 (15)	77.4 (13)	125.2 (15)
O8—C15···Cl2	3.4324 (15)	169.65 (9)	3.162 (18)	3.12 (2)	3.38 (2)	96.9 (11)	100.4 (15)	86.0 (14)
O8—C16···Cl1	3.5822 (16)	155.04 (12)	3.59 (2)	2.974 (18)	3.71 (2)	82.1 (15)	123.8 (13)	74.6 (12)

### Geometric details of contacts in 2

**Table:**

Contact	Distance (Å)
C1···C1	3.351 (12)
H2B···Li1	3.21 (8)
H2B···Li1^i^	3.24 (7)

Symmetry code: (i) -*x*+1, -*y*+1, -*z*+1.
